# Involving Adult Siblings in the Lives of Individuals With Pervasive Support Needs: Attitudes of Healthcare Professionals

**DOI:** 10.1111/jar.70139

**Published:** 2025-10-14

**Authors:** N. I. Dorsman, J. Luijkx, C. P. Van der Schans, A. A. J. Van der Putten, A. Waninge

**Affiliations:** ^1^ Research Group on Healthy Ageing, Allied Health Care and Nursing, Hanze University of Applied Sciences Groningen Groningen the Netherlands; ^2^ Inclusive and Special Needs Education, University of Groningen Groningen the Netherlands; ^3^ Department of Health Psychology University Medical Center Groningen Groningen the Netherlands; ^4^ Department of Rehabilitation Medicine University Medical Center Groningen Groningen the Netherlands

**Keywords:** attitudes, collaboration, pervasive support needs, PID, PIMD, professionals, siblings

## Abstract

**Background:**

Facilitating adult sibling involvement for individuals with pervasive support needs is important. This study explores the attitudes of healthcare professionals in this process.

**Method:**

The attitudes of healthcare professionals (*n* = 60) in the Netherlands were explored through an online, self‐developed survey with open and closed‐ended questions.

**Results:**

Around 40% of the participants reported (partly) lacking knowledge about sibling preferences and 23% (partly) lacking practical opportunities for involving siblings. The majority (partly) perceived the involvement of siblings as an enjoyable part of their work (82%), rated their knowledge and skills positively (87%), and regarded sibling involvement as such importance that they would be willing to exert considerable effort to contribute to it (61%). Not all participants perceived it as their job to collaborate with siblings.

**Conclusions:**

There is a need to increase healthcare professionals' knowledge about adult sibling preferences and structurally embed sibling involvement within care practices.

**Summary**

- Healthcare professionals play a key role in enabling sibling involvement.
- Contact with siblings is viewed as an enjoyable and important part of their jobs by many healthcare professionals.
- There is a need to increase the knowledge of healthcare professionals about sibling preferences.
- Interventions are needed to structurally embed sibling involvement within care practices.

## 1 Introduction

The importance of involvement of family members in care and support has been increasingly recognised in recent decades. Family‐centred care, originating from the field of paediatric care, has received increasing scientific attention, spreading to other healthcare settings (Hriberšek et al. 2024). Kokorelias et al. (2019) identified key components of family‐centred care that have relevance across populations with different support needs and in different settings. These key components include collaboration between healthcare professionals and family members and consideration of family contexts such as family strengths and cultural values (Kokorelias et al. 2019).

In the context of support for individuals with intellectual disabilities, researchers have explored a variety of related topics, including the parent/professional partnership (John 2020), communication between parents and healthcare professionals (Jansen et al. 2017), and the perceptions of healthcare professionals and parents regarding the family‐centred character of support services (Jansen et al. 2018). It has been acknowledged that parents possess unique experiential knowledge (Kruithof et al. 2020) and that sharing this knowledge with healthcare professionals can result in a deeper understanding of individuals with pervasive support needs (Kruithof et al. 2020), thereby making it possible to offer more customised support. The facilitation of family involvement requires good collaboration with family members and can be considered an essential aspect of the support provided to individuals with intellectual disabilities (Zambrino and Hedderich 2021). The quality of support has a major impact on the quality of life. This is especially true in the support provided to individuals with pervasive support needs and intellectual disabilities (also referred to as individuals with profound intellectual disabilities). These individuals require support 24 h a day, for all aspects of their lives and in many different domains (Nakken and Vlaskamp 2007; Van der Putten et al. 2017).

In addition to parents, it is important to involve siblings in the support of individuals with pervasive support needs and intellectual disabilities. Siblings can contribute to the quality of life of such individuals through the significant and, in many cases, multiple roles they fulfil (Bigby et al. 2015; Dorsman et al. 2023; Hall and Rossetti 2018). Some siblings fulfil, for example, the formal roles of legal representative, administrator or mentor. Examples of informal roles include the roles of advocate, personal development support or friend (Dorsman et al. 2023). In addition, siblings can contribute through the specific ways in which they interact with their siblings (Nijs et al. 2016). In many cases, siblings are the ones who stand next to parents and eventually take over (parts of) their responsibilities as parents age (Hall and Rossetti 2018; Kruithof et al. 2022; Tozer and Atkin 2015).

The involvement of adult siblings and collaboration between adult siblings and healthcare professionals is not always effortless. Adult siblings have mentioned a variety of challenges in this regard, including problems in communication and problems concerning the quality of care and support received by their siblings (Dorsman et al. 2024). Other challenges experienced by adult siblings include feelings of being ignored or judged unfairly, being expected to take the initiative for collaboration and being included only when a crisis occurs (Tozer and Atkin 2015). Negative experiences from the past apparently lead to present mistrust and low expectations (Tozer and Atkin 2015). In addition, siblings have been reported to be aware of the vulnerability of the support provided to their siblings due to organisational changes and high staff turnover (Tozer and Atkin 2015). High workloads and frequent changes of staff also pose challenges to sustainable collaboration (Dorsman et al. 2024).

Despite the importance of the topic, only limited scientific attention has been devoted to the perspective of healthcare professionals on the facilitation of sibling involvement and collaboration with siblings. Recent studies have focused on healthcare professionals working with broader target groups, that is, individuals with varied or unspecified levels of support needs and intellectual disabilities (Bigby et al. 2015; Burke et al. 2017); and on healthcare professionals serving individuals with extensive support needs, autism and learning disabilities (Tozer and Atkin 2015). Findings of these studies indicate that healthcare professionals recognise the benefits and importance of the roles of siblings and collaboration with siblings (Burke et al. 2017; Tozer and Atkin 2015). At the same time, however, healthcare providers have also been reported to struggle with the practical implementation of sibling involvement (Tozer and Atkin 2015). Siblings may be difficult to reach, and some healthcare professionals experience them as having ‘poor attitudes’, for example, being disinterested or overprotective (Burke et al. 2017). Some professionals perceive that they lack the resources needed to support the involvement of adult siblings (Burke et al. 2017; Tozer and Atkin 2015). Systemic barriers also include the lack of acknowledgement of siblings on a policy level, siblings not being included in the formal support system, and a lack of sibling support programmes (Burke et al. 2017). The quality of collaboration seems to be highly dependent on individual healthcare professionals, as there are apparently no standardised protocols relating to collaboration between healthcare professionals and family members (Bigby et al. 2015; Tozer and Atkin 2015). These findings indicate that, despite the importance of sibling involvement, it is accompanied by an array of challenges, and the facilitation of sibling involvement does not seem to be structurally embedded within practices relating to care and support.

The attitudes that healthcare professionals have towards sibling involvement constitute a key factor that affects whether and how they choose to involve siblings in specific situations. Attitude has been defined as the feelings, thoughts/beliefs and actions that create a tendency to act in a certain way (Pickens 2005). The ways in which healthcare professionals involve siblings can also be understood theoretically according to a model of behaviour. For example, the COM‐B model of behaviour describes the following three conditions: capabilities, opportunities and motivation (Michie et al. 2011). The model describes how an individual's behaviour results from these three conditions in interaction with one another. The capabilities condition refers to whether an individual is able to perform certain behaviours (Michie et al. 2011). In the example of involving siblings, this can refer to the knowledge that healthcare professionals have and their collaborative skills. The opportunity condition refers to external circumstances that influence behaviour (Michie et al. 2011), for example, whether healthcare professionals have enough time to collaborate with siblings and whether they feel supported in their efforts. The motivation condition refers to the analytical and emotional processes and habits that make an individual behave in a certain way (Michie et al. 2011). These conditions of behaviour (Michie et al. 2011) can be combined with the concept of ‘attitude’ (Pickens 2005) to explore how healthcare professionals *perceive* their capabilities, opportunities and motivation. In other words, it makes it possible to identify their feelings, thoughts/beliefs and actions concerning these three conditions. Furthermore, breaking down the general attitude towards sibling involvement into the attitudes regarding the three conditions of behaviour from the COM‐B model (Michie et al. 2011) makes it possible to draw explicit links to corresponding behavioural‐change interventions (Michie et al. 2011), thereby offering valuable suggestions for future interventions.

In summary, involving adult siblings and collaboration between healthcare professionals and siblings is accompanied by a variety of challenges from the perspectives of both healthcare professionals and siblings. Knowledge about the attitudes of healthcare professionals towards sibling involvement is fundamental to determine meaningful ways to improve collaboration and facilitate sibling involvement. To this end we sought to answer following research question: What are the attitudes of healthcare professionals in the Netherlands, concerning their capabilities, opportunities and motivation to involve adult siblings of individuals with pervasive support needs and intellectual disabilities.

## 2 Methods

### 2.1 Design

This study is based on a cross‐sectional design involving information obtained from healthcare professionals on their attitudes with regard to involving adult siblings.

### 2.2 Recruitment

Healthcare professionals with diverse occupations in terms of support for individuals with pervasive support needs and intellectual disabilities (e.g., residential support workers, daycare support workers, physiotherapists, psychologists) were recruited. Sixteen care organisations in the Netherlands were approached with the question to distribute the survey among healthcare professionals. These organisations all provide support to individuals with pervasive support needs and intellectual disabilities. In addition, participants were approached through the social media and newsletter of the academic collaborative centre where the research was carried out as well as through interest groups. Flyers were also handed out at a symposium about individuals with pervasive support needs. The criterion for inclusion in this study was that the professionals had to provide support to adults with pervasive support needs and intellectual disabilities. In line with the common terminology used within the context of healthcare in the Netherlands, the term ‘profound intellectual disability’ was used. To help individuals determine whether they were eligible to participate, the following description of ‘a profound intellectual disability’ was provided:

We speak of a profound intellectual disability when the estimated IQ is below 25 points. In this range, it is not possible to obtain reliable IQ measures. These individuals express themselves non‐verbally, for example, via sounds, facial expressions, or movements. In addition to the pervasive intellectual disability, motor or sensory impairments are often present. Support and care are needed 24 hours a day. (Adapted from Dorsman et al. 2023, 2024)

### 2.3 Data Collection

In this study, we used an online survey to explore the attitudes of healthcare professionals with regard to involving adult siblings. Survey questions were linked to the conditions from the COM‐B model of behaviour (Michie et al. 2011): capabilities, opportunities and motivation. The survey consisted of 12 closed‐ended questions. In every question, more agreement with the statement corresponded to a more positive attitude towards the respondent's capabilities, opportunities or motivation with regard to sibling involvement. For example, participants were presented with the following statement about their perceived opportunities for collaborating with siblings: ‘I think I have enough practical opportunities (e.g., time, space, information) for collaborating with adult siblings’. Thereafter, they were asked to indicate their level of agreement with this statement on a five‐point scale: completely disagree (1), partly disagree (2), neutral (3), partly agree (4) and completely agree (5). In addition to the 12 closed‐ended questions, there were 3 open‐ended questions about the participants' needs for knowledge, skills and ideas; their needs for practical opportunities and their needs for support in collaborating with siblings. Participants could type their answers to these questions in a text box with no word limit.

### 2.4 Development of the Survey

The aim of this study was to explore different aspects of healthcare professionals' attitudes with regard to involving adult siblings. The development of the survey consisted of three phases:

In the first phase, findings from previous studies on adult siblings and healthcare professionals (Dorsman et al. 2023, 2024; Tozer and Atkin 2015) were used to formulate initial questions and topics of interest.

The second phase of the development of the survey was based on an existing questionnaire that measures ‘the attitudes of direct support persons towards promoting health enhancing physical activity of persons with intellectual disabilities’ (ADSP‐HEPA) (Steenbergen 2020, 104). The ADSP‐HEPA is a brief, easy‐to‐use questionnaire with adequate internal consistency to support a one‐dimensional underlying generic factor (attitude) (Steenbergen 2020). The ADSP‐HEPA has previously been adapted to evaluate the attitudes of direct support professionals towards healthy nutrition, with promising results (Overwijk et al. 2023). This questionnaire was used because it addresses different aspects of the attitudes of care professionals and thereby structured and broadened our initial questions and topics of interest. In the current study, the questions from the ADSP‐HEPA (Steenbergen 2020) were first adapted to concern sibling involvement. Second, questions were altered and added in order to generate more detailed information and to include questions and topics from phase one. The questions about capabilities were altered to include separate questions about the knowledge of healthcare professionals concerning the information that siblings wish to receive, the ways in which they wish to be involved, and the activities that they like to do with their siblings. With regard to the questions on opportunities, we broke apart the question about the support experienced by participants, drawing a distinction between support from direct colleagues, supervisors, and the organisation. With regard to motivation, we added two questions about the job as perceived by healthcare professionals: the job to maintain contact or collaborate with adult siblings, and the job to support families in transferring tasks and responsibilities from parents to siblings as parents age. Three open‐ended questions were added to enable healthcare professionals to explain their needs in their own words.

In the third phase, the first author and co‐authors used their experiences and expertise to evaluate and adapt the survey questions. The first author was previously employed as a residential support worker, and one of the co‐authors has a professional background as a physiotherapist. These experiences enabled the research team to take the perspectives of healthcare professionals into account. The first author and co‐authors tested various versions of the survey on the online platform and discussed and adapted the questions until consensus was reached on a final version. The questions from the ADSP‐HEPA (Steenbergen 2020) and adapted questions are included in Table 1.

**TABLE 1:** Original and adapted survey questions.

Survey questions ADSP‐HEPA (Steenbergen 2020)	Domains COM‐B (Michie et al. 2011)	Adapted questions about the attitudes of healthcare professionals towards adult sibling involvement.
1. I think I am well aware of the exercise and physical activity recommendations for persons with intellectual disabilities.	Capabilities	This question concerns the wishes and needs of adult siblings of clients with pervasive support needs. I think I am well aware of: – the information they wish to receive. – the way in which they wish to be involved. – the activities they like to do with their siblings.
2. I think I have enough practical knowledge and skills to set up and carry out physical/moving activities.	Capabilities	– I think I have enough knowledge, skills, and ideas to collaborate with adult siblings of clients. *Do you need more knowledge, ideas, or skills? Would you like to explain that?*
3. I think I have enough (game) materials to carry out physical activities	Opportunities	– I think I have enough practical opportunities (e.g., time, space, information) to collaborate with adult siblings. *Do you need more practical opportunities? Would you like to explain that?*
4. I feel supported by my superiors/managers when encouraging persons with ID to be enough physically active.	Opportunities	When collaborating or being in contact with adult siblings, I feel supported by: – my direct colleagues. – my superiors. – the organisation *Do you need more support? Would you like to explain that?*
5. I think supporting physical activity is a fun part of my work.	Motivation	– I think being in contact or collaborating with adult siblings is a nice part of my work. – I think it is my job to maintain contact or collaborate with adult siblings of clients/residents. – I think it is my job to support families in transferring tasks and responsibilities from parents to siblings as parents age.
6. I believe that being physically active is so important that I will do anything to plan it in the daycare programme of persons with ID	Motivation	– I believe that the involvement of adult siblings is so important that I would exert considerable effort to contribute to it.

Background information on the participants was gathered by asking questions about their occupations and number of years working with individuals with pervasive support needs and intellectual disabilities, as well as about their age and gender.

Survey responses were collected between 25 October 2023, and 3 February 2024, using Qualtrics as the survey platform. During this period, 113 entries were recorded. Of these respondents, 23 did not provide active online informed consent and therefore did not proceed to the survey questions. Another 24 respondents indicated they did not provide support to individuals with pervasive support needs and intellectual disabilities or did not proceed beyond this question. Of the remaining respondents, six provided only background information and were therefore excluded, thus leaving 60 responses for analysis.

### 2.5 Data Analysis

Answers to the 12 closed‐ended questions were analysed according to descriptive statistics and subsequently visualised. Residential support workers and daycare support workers were distinguished from remedial educationalists/psychologists, team leaders/managers and other occupations. There were two reasons for this distinction. First, the frequency with which residential support workers and daycare support workers have contact with siblings may differ from that of other professionals. In addition, studies from adjacent fields (e.g., medical care and early‐intervention services) concerning such topics as family‐centred care, communication or collaboration with family members have previously indicated differences in attitudes, roles and behaviours across professions (Kang et al. 2017; Patel et al. 2022; Woldring et al. 2023).

Open‐ended questions were imported into Atlas‐TI and subsequently analysed according to inductive qualitative content analysis (Elo and Kyngäs 2008). This allowed us to report on the frequency of topics (Vaismoradi et al. 2013) with regard to the needs of healthcare professionals for knowledge, skills and ideas, as well as for practical opportunities and support. Coding was performed by the first author. During this process, there were regular meetings with the co‐authors to discuss the coding process and findings and to resolve any questions. Analysis consisted of the following steps:

1. An initial impression of the data was gained by reading the responses.
2. Responses were coded by assigning a code to each topic. These codes were assigned to shorter or longer fragments. Depending on the topics or ideas conveyed in the participants' answers, fragments consisted of either a few words or multiple sentences. For example, the same code was assigned to sentences that elaborated on the same topic or conveyed the same idea.
3. In the third phase, similar codes were merged, and a coding scheme was developed. This coding scheme was used to re‐code all the answers.

## 3 Results

### 3.1 Participants

Most participants (88.3%) were female, and the majority (80%) had been working with individuals with pervasive support needs and intellectual disabilities for more than 5 years. Half of the participants worked as residential or daycare support workers. Demographic information about the participants is provided in Table 2.

**TABLE 2:** Demographic data on participants.

*N* = 60 variable	*n*	%
Occupation		
Residential support worker	25	41.7
Daycare support worker	5	8.3
Remedial educationalist or psychologist	12	20.0
Team leader/manager	3	5.0
Other^a^	15	25.0
Years working with individuals with pervasive support needs and intellectual disabilities		
< 1 year	2	3.3
1–5 years	10	16.7
< 5 years	48	80.0
Age in years		
20–29	13	21.7
30–39	14	23.3
40–49	11	18.3
50–59	17	28.3
Older than 59	5	8.3

^a^ Other occupations included: speech therapists; coordinating support workers; occupational therapists; therapists; coordinators; dieticians; physiotherapists; internal supervisors/quality coordinators/team leaders and support workers in both residential and daycare settings.

### 3.2 The Attitudes of Participants With Regard to Involving Adult Siblings

**FIGURE 1:**
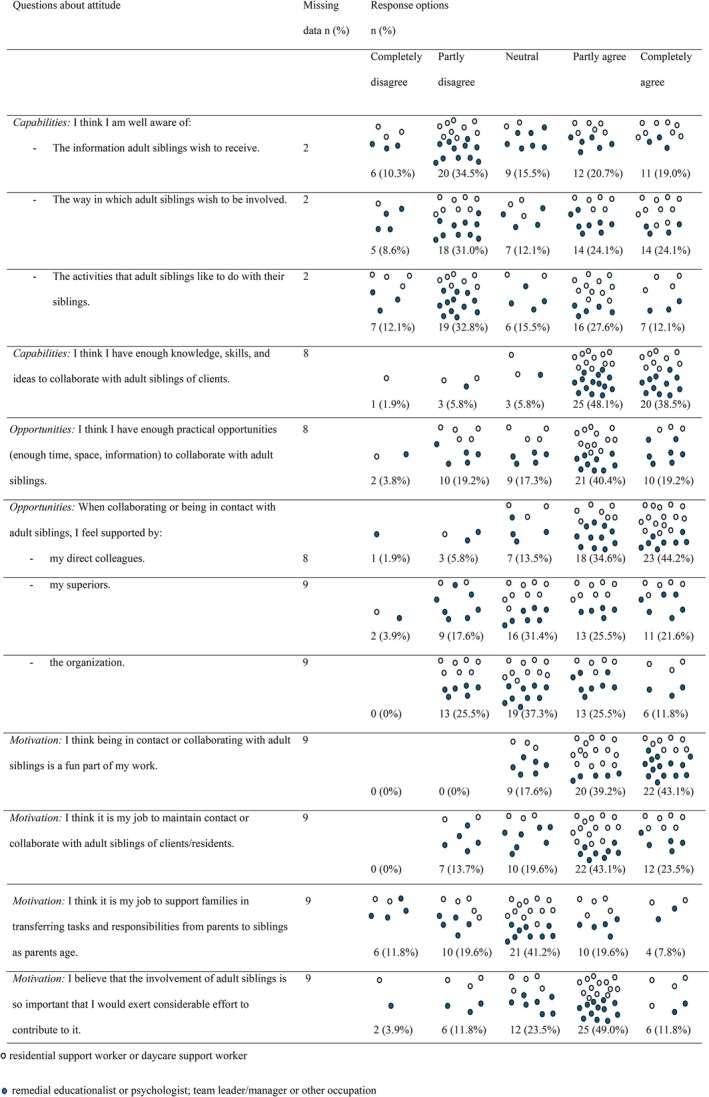
Number and percentages of responses to each of the questions about attitude.

For the 12 close‐ended questions, the number and percentages of the answers assigned to each of the response options are presented and visualised in Figure 1. With regard to perceived capabilities, 86.6% of the participants assigned positive ratings to their knowledge, ideas and skills for collaborating with siblings. Mixed ratings were assigned to their knowledge about the preferences of siblings, with 44.8% assigning negative scores to their knowledge about the information siblings wish to receive, 39.7% assigning a negative rating to their knowledge about how siblings would like to be involved, and 44.8% assigning a negative rating to their knowledge about the activities that siblings like to do with their siblings with support needs.

With regard to perceived opportunities, more than half of the participants assigned a positive rating to the practical opportunities for collaborating and 23.1% assigned a negative rating. In addition, as indicated in Figure 1, participants perceived receiving more support from direct colleagues than from supervisors or the organisation.

With regard to motivation, all participants at least partly agreed with the statement that collaboration with siblings is a enjoyable part of their work. Not all participants considered it to be part of their jobs. Answers to the question whether professionals regard it as their job to support families in transferring roles from parents to siblings were mixed. More than half of the participants (60.8%) at least partly agreed with the statement: ‘I think that the involvement of adult siblings is so important that I would exert considerable effort to contribute to it’.

### 3.3 Needs for Capabilities and Opportunities

The open‐ended questions were answered by 33 participants. Two topics stood out with regard to their needs: the limited amount of time available and the limited contact with siblings. Furthermore, participants mentioned additional challenges and needs in relation to collaboration with siblings.

#### 3.3.1 Limited Time and Limited Contact

As a first main topic, participants reported having only limited time for contact and collaboration with siblings or expressed a desire for more time to collaborate (*n* = 15, of whom 8 are residential or daycare support workers, 4 are remedial educationalists or psychologists, 1 is a team leader/manager and 2 are participants with other occupations).

Time is already scarce in these groups. I therefore don't see that we get enough leeway for this. (Participant 1, daycare support worker)

Outside client time, we have almost no time for contact with family. I do this a lot in my own time. (Participant 27, daycare support worker)

It often requires an extra investment of time, which with the current shortages is not always available. (Participant 48, speech therapist)

Time. Now often only once a year during the year evaluation. (Participant 49, remedial educationalists/psychologist)

The second main topic concerned the limited contact with siblings or the desire for more contact with siblings, as indicated by 17 participants (6 residential or daycare support workers, 6 remedial educationalists or psychologists and 5 participants with other occupations). They described several reasons for the limited contact. For example, some therapists noted that, within their organisations, therapists had no contact with family members or that such contact proceeded through direct care professionals. Within this context, one therapist described not wanting to impose any unnecessary burden on family members. A daycare support worker remarked that siblings only visited the group home, which resulted in less contact with healthcare professionals working in daycare settings. Some participants noted that their contact tended to be directed primarily towards parents or that the initiative to have contact was left to the siblings:

Young adults are living in the group home where I work. On average, the parents are in their 50s, so the care, communication, and contact is mainly with parents. Siblings are not as visible. (Participant 37, residential support worker)

The opportunities are there. I just often don't actively involve them if they don't do it on their own accord. (Participant 2, remedial educationalist/psychologist)

Participants also mentioned that siblings are busy or that they live far away. A remedial educationalist/psychologist expressed a desire to involve siblings before the phase in which they take over roles from parents. As described by this participant, parents regularly make a conscious choice not to burden their children with the care and support for their sibling.

#### 3.3.2 Other Challenges and Needs Relating to Collaboration With Siblings

Four participants reported that the involvement of siblings received only limited attention within their organisations and that the relationships with relatives should be strengthened:

We never talk about that with either the manager or the organization. (Participant 56, remedial educationalist/psychologist)

Things go the way they go. I haven't thought about this before. Interesting point for a meeting. These contacts are important; can we help to strengthen the ties? (Participant 38, residential support worker)

Other factors (each mentioned by one participant) included unclear responsibilities regarding collaboration with family members; the fact that coordination is more complicated when more people are involved; insufficient enthusiasm for the way things were organised at the time and not regarding collaboration or communication with siblings as a responsibility of a direct care professional. One participant also stated that the organisation should include siblings in the distribution of information about activities and events and another participant mentioned the need to support siblings (e.g., by organising days for relatives).

Whereas several participants mentioned having sufficient knowledge, skills, or support regarding sibling involvement, seven others indicated that they lacked knowledge about the wishes of siblings or expressed a desire to know more about the experiences of siblings:

I would like to have more time for relatives. And more knowledge about what they are going through or have gone through. (Participant 39, residential support worker)

Where I work, adult siblings are not very involved. I don't know whether this is also what they want, or whether there might be a desire for more contact, of which I, as a care worker, am not aware. (Participant 32, residential support worker)

Three participants mentioned needing better collaborative skills, especially in situations where the relationship was strained. Other participants mentioned needing more experience, more practical information about sibling involvement or more support (*n* = 1 each). Two participants described digital communication as helpful in maintaining contact with siblings.

## 4 Discussion

In this study, healthcare professionals who provided support to individuals with pervasive support needs and intellectual disabilities were asked about their attitudes with regard to involving siblings in relation to three conditions of behaviour: perceived capabilities, opportunities, and motivation (Michie et al. 2011). With regard to their perceived capabilities, most participants were positive about their own knowledge, ideas, and skills relating to collaboration. They assigned lower ratings to their knowledge about the preferences of siblings (i.e., the information they wish to receive, how they would like to be involved, and what activities they like to do with their siblings), with 40%–45% of participants indicating that they at least partly lacked this knowledge. In addition, a substantial share of the participants expressed a need for practical opportunities for collaborating with siblings. This was also reflected in the answers to the open‐ended questions, in which limited time and limited contact were the most common themes. With regard to their contact with siblings, participants experienced receiving more support from direct colleagues than from supervisors or the organisation. With regard to their motivation, many participants viewed contact with siblings as an enjoyable part of their jobs, and more than half indicated that sibling involvement is so important that they would exert considerable effort to contribute to it. Not all participants considered it their job to collaborate with siblings or to provide support to families in transferring roles from parents to siblings as parents age.

The results of the current study show that most participants perceived they had sufficient knowledge, ideas, and skills, even as many of them at least partly lacked knowledge about the preferences of siblings. This aligns with previous observations that healthcare professionals struggle with the practical implementation of sibling involvement (Tozer and Atkin 2015). This lack of knowledge about the preferences of siblings may also be linked to their experiences of feeling ignored or excluded (Tozer and Atkin 2015). Relatives who were involved as representatives have described the importance of being involved and of being acknowledged in their roles (Koelewijn et al. 2023). Knowledge about roles, experiences, and preferences is crucial to the acknowledgement of relatives—both those who fulfil formal roles and those who are involved in the lives of their siblings in other ways. One possible explanation for the discrepancy between the perceived collaborative skills of healthcare professionals and their lack of knowledge of sibling preferences could be that the participants overestimated their skills. Although healthcare professionals may feel that they have enough knowledge, skills and ideas to collaborate, in practice, they may not succeed in building the collaborative relationships with siblings that would enable them to gain knowledge about their preferences. A second possible explanation is that collaboration with siblings is complicated by other factors (e.g., a lack of practical opportunities). According to the findings of this study, healthcare professionals do indeed face considerable challenges with regard to the practical opportunities for collaborating with siblings. The lack of available time for collaboration was a particularly striking factor. Staffing shortages, high workload, and a lack of available time constitute widely acknowledged challenges in the provision of support to individuals with disabilities (Wagenaar and Coenders 2021; Overwijk et al. 2021; Zambrino and Hedderich 2021). In addition, challenges relating to the time available for collaborating with family members are not unique to the provision of support to individuals with intellectual disabilities. For example, within the context of care for individuals with chronic conditions, Hengelaar et al. (2018) describe how support for informal caregivers tends to be provided informally and in spare time.

Participants in the current study rated collaboration with siblings as either a neutral or positive part of their jobs, and not all participants regarded it as part of their duties. This is in line with the findings of Bigby et al. (2015) concerning an apparent lack of certainty in group home services about who is responsible for the relationships with siblings. Notably, only about a quarter of the healthcare professionals in the current study considered it their job to support families in transferring roles from parents to siblings as parents age. It has been widely argued that it is important to support families in the complex and emotionally charged process of discussing and planning for the future (Kruithof et al. 2021; Leane 2020; Davys et al. 2016; Burke et al. 2012, 2020). Specific intervention programmes aiming at such transitions are needed.

## 5 Limitations and Strengths of the Study

The results of the current study provide insight into the attitudes of healthcare professionals with regard to involving siblings, thereby offering suggestions for future interventions. The COM‐B model of behaviour (Michie et al. 2011) provided a useful framework for the development of the survey. The distinction between capabilities, opportunities and motivation ensured that various aspects of the attitudes of healthcare professionals were taken into account. In addition, the link with possible intervention functions increased the practice‐oriented focus of this study.

Several important limitations of this study need to be mentioned. Despite the fact that participants were recruited through different healthcare providers and various social media channels, their distribution across healthcare providers remains unknown, as the responses were recorded anonymously. We, therefore, cannot rule out the possibility of a bias due to the over‐representation or under‐representation of certain healthcare providers, with specific local cultures and ways of working with regard to sibling involvement. Healthcare professionals' attitudes and practices in collaborating with siblings may also differ across countries. In addition, healthcare professionals who had more interest in collaborating with family members may have been more motivated to respond to the survey, possibly resulting in a bias towards more positive attitudes with regard to sibling involvement. This possible response bias should be considered when attempting to generalise the findings of this study. Another limitation is the possibility that social desirability played a role in the answers regarding attitudes (Steenbergen 2020), thereby introducing a bias towards more positive attitudes. Furthermore, the sample size was limited. It is important to note, however, that a small sample size was expected, as the survey targeted healthcare professionals who provide support to a small and specific target group (Maes et al. 2021).

We visually compared the responses of healthcare professionals working as residential support or daycare support workers to those of remedial educationalists/psychologists/team leaders/managers/other occupations. Responses to the open‐ended questions indicated that, for therapists in some facilities, contact with family members proceeded through direct care professionals. The heterogeneity with regard to the participants' occupations may have influenced the results and a larger sample may have made it possible to investigate these differences statistically. Finally, the survey contained general questions with regard to involving adult siblings. However, participants' experiences may have differed across situations. The questions did not provide the opportunity to specify how their experiences in collaboration differed according to the roles that siblings fulfilled, for example, formal or informal roles, or the siblings' level of involvement.

## 6 Implications for Research and Practice

Expanding on the findings of this explorative study, it would be valuable to gain more knowledge about possible differences in attitudes between professions concerning the involvement of siblings. It would also be valuable to further explore if healthcare professionals agree about whose responsibility it is to involve siblings. In addition, healthcare professionals' experiences of involving siblings may differ according to the formal or informal roles that siblings fulfil. The limited awareness reported by the participating healthcare professionals, with regard to sibling preferences, is striking. The regular communication that is essential for rewarding collaboration between healthcare professionals and family members (Zambrino and Hedderich 2021) appears to be lacking between some healthcare professionals and siblings. This can have serious consequences for collaboration. A special focus should be directed towards identifying the wishes of siblings in terms of both collaboration and their involvement in the lives of their siblings. Awareness about sibling preferences could enable healthcare professionals to adapt their collaborative efforts, thereby making the collaborative relationship more fulfilling for both parties. It might also help siblings to feel more acknowledged in the roles they fulfil. When healthcare professionals take an active role and share the information siblings need in order to be involved (e.g., concerning the day programme or activities that siblings could join), this can have an empowering effect, as it creates opportunities for involvement on an equal basis.

The range of intervention functions targeting the capabilities of healthcare professionals could include education, training and enablement (Michie et al. 2011). As suggested by Hengelaar et al. (2018), educational curricula in the fields of social work, allied health and nursing should be adjusted to more extensively address the support for informal caregivers. This is particularly important in light of the challenges that the healthcare system is currently facing, including the growing personnel shortages (Helder 2023), which are likely to increase the need for close collaboration with the informal social network of individuals with support needs. It is highly recommended that future healthcare professionals should receive explicit training on siblings as members of the informal networks of their clients. Possible topics could include the roles fulfilled by siblings, their experiences, how these roles and experiences change over the life course and how their involvement can contribute to the quality of life of individuals with support needs. Current healthcare professionals could also benefit from education on this topic (e.g., through e‐learning modules, educational magazines or short films). Such education could contribute to siblings being more ‘in the picture’. Depending on their wishes, this could, for example, result in siblings receiving information, being invited to visit their sibling, to join festivities, or to participate in service planning meetings.

Guidelines should be developed to incorporate the creation of an inventory of the wishes and needs of adult siblings as a standard and recurring element of the individualised support planning. Such inventories are essential, given that the roles and experiences of siblings change over the life course (Hall and Rossetti 2018; Tozer and Atkin 2015). Siblings who have only limited contact during a specific period may have different wishes a few years later. An open and recurring conversation about wishes and needs could also help siblings feel recognised and understood in their continuing attempts to balance involvement with their siblings and other aspects of their lives (Dorsman et al. 2024; Tozer and Atkin 2015). Intervention functions focusing on ‘environmental restructuring’ (Michie et al. 2011) could assist healthcare professionals in their efforts to involve siblings. One option could be to restructure the digital care file to include the topic of sibling involvement (e.g., by providing on‐screen prompts). If a physical care file is used, a card with information about the contact details and preferences of siblings could be added (Tozer et al. 2013). Special attention should be directed towards the support of families in the transferring of roles from parents to siblings as parents age. Another area that deserves more attention has to do with the division of roles between healthcare professionals. It should be clear what responsibilities various professionals (i.e., residential support workers, daycare support workers, therapists, remedial educationalists/psychologists or team leader/managers) have in building and maintaining collaborative relationships with siblings and other family members.

The love, social interactions, knowledge and advocacy that some siblings offer (Dorsman et al. 2023, 2024) can have a positive effect on the quality of life of individuals with pervasive support needs. For individuals living in residential care facilities, therefore, it is extremely important for their siblings to be enabled and supported in their efforts to be involved, according to their wishes. Healthcare professionals play a key role in enabling sibling involvement. The results of this study suggest that future interventions should be aimed at both changes in the system and the education of healthcare professionals.

## Ethics Statement

Ethical approval for this interview study was obtained from the Ethical Review Board of the Hanze University of Applied Sciences Groningen (approval number: heac.T2023.030.). Participation in the study was voluntary, and responses were anonymous. Participants provided informed consent by checking a box at the start of the survey.

## Consent

All participants provided active online informed consent.

## Conflicts of Interest

The authors declare no conflicts of interest.

## Data Availability

The data that support the findings of this study are available on request from the corresponding author. The data are not publicly available due to privacy or ethical restrictions.
